# Antiphospholipid antibody‐activated NETs exacerbate trophoblast and endothelial cell injury in obstetric antiphospholipid syndrome

**DOI:** 10.1111/jcmm.15321

**Published:** 2020-05-05

**Authors:** Yuan Lu, Yan Dong, Yan Zhang, Di Shen, Xiyao Wang, Ruxiu Ge, Meihua Zhang, Yu Xia, Xietong Wang

**Affiliations:** ^1^ Department of Obstetrics and Gynaecology Shandong Provincial Hospital Affiliated to Shandong University Jinan China; ^2^ Department of Obstetrics and Gynaecology Linyi People’s Hospital Linyi China; ^3^ Department of Obstetrics and Gynaecology Maternal and Child Health Care of Shandong Province Jinan China; ^4^ The Laboratory of Placenta‐Related Diseases Key Laboratory of Birth Regulation and Control Technology of National Health and Family Planning Commission of China Jinan China; ^5^ Department of Central Laboratory Shandong Provincial Hospital Affiliated to Shandong University Jinan China

**Keywords:** antiphospholipid syndrome, endothelial cell, neutrophil extracellular trap, obstetric APS, trophoblast

## Abstract

Despite the widespread use of antiplatelets and anticoagulants, women with antiphospholipid syndrome (APS) may face pregnancy complications associated with placental dysplasia. Neutrophil extracellular traps (NETs) are involved in the pathogenesis of many autoimmune diseases, including vascular APS; however, their role in obstetric APS is unclear. Herein, we investigated the role of NETs by quantifying cell‐free DNA and NET marker levels. Live‐cell imaging was used to visualize NET formation, and MAPK signalling pathway proteins were analysed. Cell migration, invasion and tube formation assays were performed to observe the effects of NETs on trophoblasts and human umbilical vein endothelial cells (HUVECs). The concentrations of cell‐free DNA and NETs in sera of pregnant patients with APS were elevated compared with that of healthy controls (HCs) matched to gestational week. APS neutrophils were predisposed to spontaneous NET release and IgG purified from the patients (APS‐IgG) induced neutrophils from HCs to release NETs. Additionally, APS‐IgG NET induction was abolished with inhibitors of reactive oxygen species, AKT, p38 MAPK and ERK1/2. Moreover, NETs were detrimental to trophoblasts and HUVECs. In summary, APS‐IgG‐induced NET formation deserves further investigation as a potential novel therapeutic target in obstetrical APS.

## 1 INTRODUCTION

Antiphospholipid syndrome (APS) is an autoimmune disease characterized by persistently elevated titres of antiphospholipid antibodies (aPLs) that predispose individuals to arterial and/or venous thrombosis or to pregnancy complications^1^, ^2^; the latter includes recurrent spontaneous abortion, pre‐mature birth caused by pre‐eclampsia and foetal growth restriction. Although the pathogenesis of these pregnancy complications is still not fully understood, it has been suggested to be a consequence of intraplacental thrombosis^3^, ^4^; however, increasing studies have suggested that placental inflammation is a hallmark characteristic of adverse pregnancy outcome complicated by aPL.^4^ Current treatments for APS focus on inhibiting coagulation rather than treating the potential pathophysiology.^5^

Antiphospholipid antibodies are a group of heterogeneous autoantibodies that bind a complex antigen involving phospholipid‐binding proteins, such as β2‐glycoprotein I (β2GPI), and negatively charged phospholipids, such as cardiolipin.^6^ Antiphospholipid antibodies induce thrombosis by activating endothelial cells and platelets and impede placentation by damaging trophoblasts directly. Furthermore, activated neutrophils in APS may play an important role in foetal loss.^7^ Circulating neutrophils from patients with APS are in an activated state, as indicated by an associated increase in the production of reactive oxygen species (ROS).^8^ In a mouse model of obstetric APS, Girardi and colleagues^9^ observed a significant inflammatory response in decidual tissue with a large number of neutrophils present in vivo. In this mouse model, depleting mice of neutrophils or inhibiting complement factor C5a can eliminate the deleterious effects of aPLs,^9^, ^10^ indicating that both neutrophils and the complement system have important pathophysiological effects in APS.^9^

Neutrophil extracellular traps (NETs) are structures made of chromatin scaffolds covered with histones, proteases, granules and cytosolic proteins. NETosis describes the process by which neutrophils produce and release NETs.^11^ NET formation occurs both in infectious diseases and in non‐pathogenic conditions, such as autoimmune diseases. Low‐density granulocytes are an inflammatory subset of neutrophils in systemic lupus erythematosus (SLE), which demonstrate robust demethylation of interferon genes^12^ and release excessive NETs.^13^ NET‐mediated endothelial cell toxicity has been observed in patients with lupus nephritis,^13^ and NETs may induce endothelial cell apoptosis and promote vascular injury in murine lupus models.^14^, ^15^ Excessive NET formation and the incomplete degradation of NETs have been considered to contribute to the pathogenesis of anti‐neutrophil cytoplasmic antibody (ANCA)‐associated vasculitis (AAV).^16^ Moreover, the rheumatoid arthritis (RA) synovial microenvironment is highly conducive to the release of NETs with the potential to activate immune cells in the synovium; thus, targeting NET formation as a novel treatment strategy may improve the prognosis of RA patients.^17^ On the other hand, in bladder cancer treatment with Bacillus Calmette‐Guerin, NETs were found to exert cytotoxicity, induce apoptosis and cell‐cycle arrest, and inhibit the migration of bladder tumour cells.^18^

Yalavarthi et al^19^ reported that incubation with aPL stimulates neutrophils from healthy volunteers to release NETs and that neutrophils in patients with APS are more prone to NETosis than are healthy volunteer neutrophils. As NETs stimulate platelet activation and thrombosis^20^ and are present in placentas of patients with pre‐eclampsia,^21^ strong exposure to NETs may be related to the pathogenesis of APS. However, there are no studies reporting whether neutrophils from pregnant women with APS are more prone to NETosis, and, aside from the influence of NETs on thrombosis, whether they cause direct damage to umbilical vein endothelial cells and trophoblasts.

Therefore, we investigated aPL/neutrophil interplay in pregnant women with APS and examined the effects of NETs on umbilical vein endothelial cells and trophoblasts, with the aim of improving the prognosis of pregnant women with APS.

## 2 MATERIALS AND METHODS

### 2.1 Patient information

Given the established links between NETs and autoimmune diseases and that are some differences between obstetric APS and vascular APS, we focused on pregnant women with APS for the main experiments of our study. Written informed consent was obtained from all patients. This study was carried out according to the World Medical Association Declaration of Helsinki and was approved by the ethics committee of Shandong Provincial Hospital affiliated to Shandong University.

Blood from individuals with a diagnosis of APS or from healthy controls (HCs) was obtained at Shandong Provincial Hospital affiliated to Shandong University. All patients met the APS criteria, which were updated in Sydney in 2006.^1^ All 22 pregnant patients with APS had pregnancy‐related morbidities but no thrombosis, and none met the American College of Rheumatology criteria for SLE.^22^ Patients with infections, other inflammatory disorders or malignancies were excluded because of a potentially increased formation of NETs. Pregnant women who were negative for aPLs, had no associated pregnancy complications and had experienced at least one healthy pregnancy were chosen as our HCs. Antibody detection in patients and HCs was carried out at Shandong Provincial Hospital affiliated to Shandong University. Anti‐β2 glycoprotein I (anti‐β2GP‐I) antibodies and anti‐cardiolipin antibodies were quantified using enzyme‐linked immunosorbent assay (ELISA) detection kits (Euroimmun, Lübeck, Germany), whereas lupus anticoagulant (LA) was quantified via the LA1 screening reagent and LA2 confirmation reagent (Siemens Healthineers, Erlangen, Germany). The details of all recruited participants are presented in Table 1, and gestation weeks are listed in Table S1.

**TABLE 1:** Characteristics of patients enrolled in this study

Characteristic	APS	HC
Number (n)	22	22
Age (y)*	30 (21‐34)	29 (21‐34)^n.s.^
Gestational weeks (wk)	12.19 (11‐13^+4^)	12.47 (11 + 13^+6^)^n.s.^
Clinical manifestations		
Recurrent spontaneous abortions	18 (82%)	0
Severe pre‐eclampsia	5 (23%)	0
Unexplained stillbirth	2 (9%)	0
Oligohydramnios	1 (4%)	0
Foetal growth restriction	1 (4%)	0
Lupus anticoagulant positive (% patients)	16 (73%)	0
Anti‐β2GPI IgG	22 (100%)	0
Anti‐β2GPI IgM	6 (27%)	0
Anti‐cardiolipin IgG	11 (50%)	0
Anti‐cardiolipin IgM	4 (18%)	0

Abbreviations: β2GPI, β2‐glycoprotein I;APS, antiphospholipid antibody syndrome; HC, healthy control; n.s., not significant (*t* test).

* Median (range).

### 2.2 **Quantification of neutrophil elastase, myeloperoxidase, cell‐free DNA and **myeloperoxidase**‐DNA complexes**

Human whole blood from patients and healthy volunteers was collected into tubes containing no anticoagulants to obtain sera. Neutrophil elastase (NE) and myeloperoxidase (MPO) levels in sera were detected using the respective ELISA kits (Abcam, Cambridge, UK) according to manufacturer's instructions. Cell‐free DNA in sera was quantified using the Quant‐iT PicoGreen dsDNA Assay Kit (Invitrogen, Carlsbad, CA) according to manufacturer's instructions. Quantifying MPO‐DNA complexes was performed as previously described^23^, ^24^ using several reagents from the Cell Death Detection ELISA Kit (Roche, Basel, Switzerland). Briefly, the anti‐human MPO antibody (ab25989; Abcam) was diluted to a concentration of 5 µg/mL in coating buffer (provided in the kit) and used to coat a Costar high‐binding EIA/RIA 96‐well plate (Corning Inc, Corning, NY) overnight at 4°C. The plate was blocked with incubation buffer for 90 minutes at room temperature, washed three times with wash buffer and then incubated overnight at 4°C with 20% sera in incubation buffer. The plate was washed four times and then incubated with 1X anti‐DNA antibody (HRP‐conjugated; provided in the kit) diluted in incubation buffer for 90 minutes at room temperature. After three washes, the plate was developed with the peroxidase substrate (ABTS) provided in the kit. The absorbance at a wavelength of 405 and 490 nm was measured using a Synergy HT Multi‐Mode Microplate Reader (Bio‐Tek, Winooski, VT) after 40 minutes of incubation at 37°C in the dark.

### 2.3 Purification of patient immunoglobulin G (IgG)

IgG was purified from APS or control sera with a NAb™ Protein A Plus Spin Kit (Thermo Fisher Scientific, Waltham, MA) according to manufacturer's instructions and as previously described.^24^ Briefly, sera were passed through a Protein A Agarose Column at least three times. IgG was then eluted with 0.1 mol/L glycine and neutralized with 1 mol/L Tris. IgG purified from APS sera was termed APS‐IgG. IgG purified from control sera was termed HC‐IgG. IgG concentrations were determined by a BCA protein assay (Solarbio, Beijing, China) according to manufacturer's instructions. IgG purity was verified with Coomassie staining. All IgG samples were determined to contain no detectable endotoxins using a Chromogenic Endotoxin Quantitation Kit (Thermo Fisher Scientific) according to manufacturer's instructions.

### 2.4 Neutrophil isolation

Human whole blood from patients and healthy volunteers was collected into ethylenediaminetetraacetic acid (EDTA)‐containing tubes. Neutrophils were isolated by density gradient centrifugation using Polymorphprep™ (Axis‐Shield, Dundee, Scotland) according to manufacturer's instructions. Neutrophils were resuspended in Roswell Park Memorial Institute (RPMI) 1640 medium (phenol red‐free; Gibco; Thermo Fisher Scientific) supplemented with 2% foetal bovine serum (FBS; Gibco) and cultured at 37°C and 5% CO_2_. Neutrophil purity was >90%, as determined by flow cytometry using CD15‐FITC (BD Biosciences, Franklin Lakes, NJ) and cytomorphology. Cell viability was >90%, as determined by trypan blue (Solarbio) exclusion.

### 2.5 NET production

Costar culture plates (Corning Inc) were coated with 100 µg/mL poly‐L‐lysine (Solarbio) according to manufacturer's instructions before freshly isolated neutrophils (1 × 10^7^ cells/mL) were gently added. After incubation at 37°C in 5% CO_2_ for 0.5‐1 hours, neutrophils were stimulated with APS‐IgG (15 µg/mL), HC‐IgG (15 µg/mL) and phorbol‐12‐myristate‐13‐acetate (PMA; 50 nmol/L; Sigma‐Aldrich, St. Louis, MO) or left untreated.

### 2.6 Cell‐free NET purification

To purify cell‐free NETs, 2 × 10^6^ cells were added into 6‐well plates, incubated and stimulated as described above. Following stimulation for 4 hours, cells were gently washed after the medium was removed. After addition of 500 µL RPMI (phenol red‐free) to the adherent film and vigorous agitation, the samples were centrifuged at 2000 × *g* for 5 minutes and the supernatant collected as previously described.^25^, ^26^, ^27^ Cell‐free DNA and protein levels were quantified using the Quant‐iT PicoGreen dsDNA Assay Kit and a BCA protein assay, respectively, according to manufacturers’ instructions.

### 2.7 Live‐cell imaging

Neutrophils (2 × 10^5^ cells/mL) were seeded into black 96‐well plates in 200 µL RPMI 1640 (phenol red‐free) supplemented with 2% FBS. Cells were then stimulated with APS‐IgG (15 µg/mL), HC‐IgG (15 µg/mL) and PMA (50 nmol/L) or left untreated for 2 hours. NETs were detected using a mixture of cell‐permeable (Hoechst 33342; Thermo Fisher Scientific) and cell‐impermeable (SYTOX Green; Invitrogen) fluorescent DNA dyes. Samples were imaged using a high content screening microscope (ImageXpress Micro Confocal; Molecular Devices, San Jose, CA) with MetaXpress software. For NET quantification, at least five images were randomly taken from different regions of the wells (three repeats for each condition). Cell counts were determined by ImageJ software (US National Institutes of Health, Bethesda, MD), and the per cent of NETs formed was calculated as follows: (number of cells showing NETosis/total number of cells) × 100.

### 2.8 Immunofluorescence

Neutrophils (8 × 10^4^) were seeded into black 96‐well plates coated with poly‐l‐lysine (Sigma‐Aldrich) and allowed to adhere for 30‐60 minutes before stimulation with APS‐IgG (15 µg/mL), HC‐IgG (15 µg/mL) and PMA (50 nmol/L) or left untreated for 2.5 hours. Cells were then fixed in 4% paraformaldehyde without permeabilization and blocked with goat serum (Boster, Wuhan, China) at 37℃ for 1 hour. NETs were detected with a mouse monoclonal primary anti‐MPO antibody (ab25989; Abcam) diluted 1:200 in blocking buffer overnight at 4℃. Next, the plates were incubated with 1:500 rhodamine‐conjugated anti‐mouse IgG (ZsBio, China) for 1 hour at room temperature, followed by DNA counterstaining with 4′,6‐diamidino‐2‐phenylindole (DAPI; Solarbio). Samples were imaged using ImageXpress Micro Confocal with MetaXpress software. NETs were counted and expressed as a percentage of the total number of cells in the fields.

### 2.9 Signalling pathway detection via Western blotting

Neutrophils treated with APS‐IgG (15 µg/mL), HC‐IgG (15 µg/mL) or PMA (50 nmol/L), as mentioned above, were lysed with radio‐immunoprecipitation assay lysis buffer (Beyotime, Beijing, China) on ice for 1 hour. Protein concentration was quantified (after spinning to remove debris) using the BCA protein assay. Primary antibodies used were as follows: phospho‐Akt (p‐Akt), Akt, phospho‐p44/42MAPK (ERK1/2, Thr202/Tyr204), p44/42 MAPK (ERK1/2), phospho‐p38 MAPK (Thr180/Tyr182) and p38 MAPK; all were purchased from Cell Signaling Technology (Danvers, MA). HRP‐conjugated goat anti‐rabbit antibody (Proteintech, Rosemont, IL) was used as secondary antibody. The blots were then developed using Immobilon Western Chemiluminescent HRP Substrate (Merck Millipore, Burlington, MA) and imaged using the Amersham Imager 600 Imaging System (GE Healthcare, Chicago, IL); relative densitometric quantification was performed with ImageJ.

### 2.10 Isolation of human umbilical vein endothelial cells

We chose human umbilical vein endothelial cells (HUVECs) as a endothelial cell model for our in vitro experiments. We obtained human umbilical cords from the Department of Obstetrics and Gynecology of Shandong Provincial Hospital affiliated to Shandong University. The umbilical cords were treated within 2 hours after collection. All umbilical cords were derived from full‐term, single‐foetus pregnancies without complications. Briefly, the umbilical cord vein was washed twice using phosphate‐buffered saline (PBS) containing 1% penicillin and 1% streptomycin (Solarbio). The vein was then injected with 0.25% trypsin in EDTA through a gavage needle after both ends of the vein were clamped with haemostats. After 10 minutes, the digestion was stopped with complete endothelial cell medium (ECM) supplemented with 20 mg/mL endothelial cell growth supplement, 1% penicillin‐streptomycin and 10% FBS. The vein was additionally washed three times with PBS containing 1% penicillin and 1% streptomycin. After centrifugation at 180 *g* for 10 minutes, the cells were resuspended in complete ECM.

### 2.11 Cell culture

The first‐trimester human extravillous trophoblast cell line HTR‐8/SVneo was purchased from the American Type Culture Collection (ATCC; Manassas, VA). The cells were maintained in RPMI 1640 medium supplemented with 10% FBS and 100 nmol/L penicillin/streptomycin (Gibco). Neutrophils and HUVECs were isolated as mentioned above. All cells were maintained in a humidified chamber at 37°C and 5% CO_2_.

### 2.12 Cell migration and invasion

Cell migration and invasion were measured using a two‐chamber Transwell migration assay, as previously described.^28^ The lower chamber (24‐well plate) was filled with 600 μL of complete medium. HTR‐8/SVneo trophoblast cells or HUVECs (1 × 10^4^ in 200 μL RPMI 1640 medium for migration and 3 × 10^4^ in 200 μL RPMI 1640 medium for invasion; media were supplemented with 2% FBS and 100 nmol/L penicillin/streptomycin) with different treatments were seeded onto cell culture well inserts with an 8‐μm pore size membrane (Corning Inc). The non‐migrated cells were removed from the upper chambers with a cotton‐tipped swab after 24 hours. Cells that migrated or invaded across the membrane were fixed with 4% paraformaldehyde and stained with crystal violet dye. Finally, the average number of migrated or invaded cells was counted using an inverted microscope (Olympus, Tokyo, Japan) at a magnification of 100×. For invasion assays, the upper chamber membrane was pre‐coated with 60 μL of Matrigel mix (BD Bioscience) at 37°C overnight.

### 2.13 Scratch wound‐healing assay

HTR‐8/SVneo cells (5 × 10^5^) were seeded in a 6‐well plate and cultured under a humidified atmosphere of 5% CO_2_ at 37°C. A clean scratch across the centre of the cell layer was generated with a p1000 sterile pipette tip after the cells had formed a confluent monolayer. Floating cells were gently washed away with PBS. Subsequently, cells were treated with NETs or medium and then imaged after 24 hours (4× objective). Cell migration area was estimated using ImageJ.

### 2.14 Tube formation

Matrigel was diluted with ECM at a 1:1 ratio and added to 96‐well plates. The plates were placed in a 37°C incubation room for 30 minutes to allow the Matrigel to polymerize. HUVECs were seeded onto the Matrigel‐coated wells at 1 × 10^4^ cells/well and incubated for 6 hours at 37°C in growth supplement‐free medium in the presence of the indicated test compounds. Samples were visualized using an inverted microscope, and ImageJ was used to quantify tube formation.

### 2.15 Statistical analysis

Data are expressed as means ± SEM of three independent experiments. Statistical differences between samples were assessed by Student's *t* test or one‐way ANOVA. Statistical analysis was performed with GraphPad Prism (ver. 7; GraphPad Software Inc, La Jolla, CA). *P* values < 0.05 were considered statistically significant.

## 3 RESULTS

### 3.1 Sera from pregnant women with APS show increased cell‐free DNA and NETs

**FIGURE 1:**
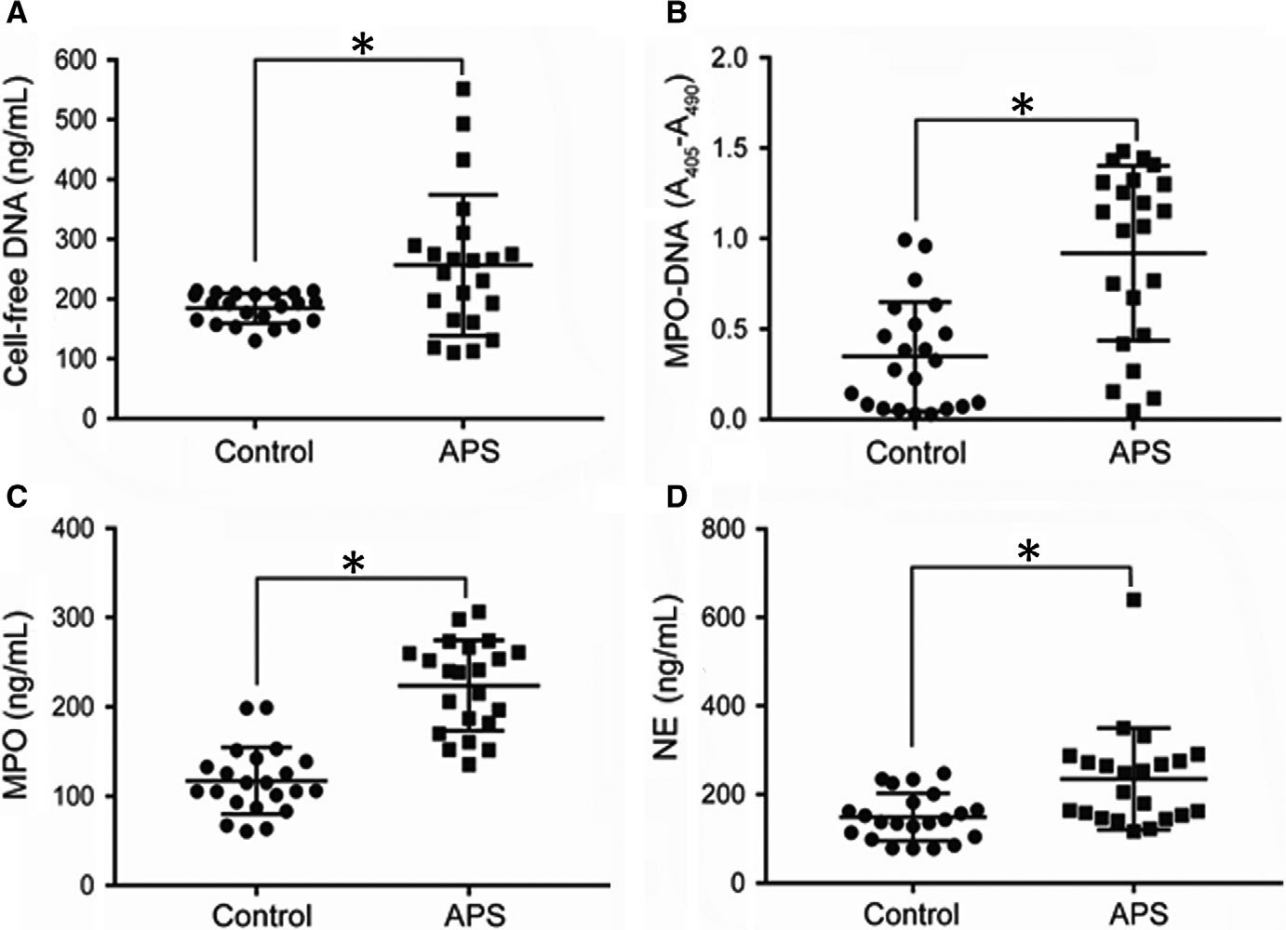
Sera of pregnant women with antiphospholipid syndrome (APS) exhibit increased cell‐free DNA and neutrophil extracellular trap (NET) levels. A, Cell‐free DNA was measured in the sera of pregnant women with APS (n = 22) or healthy control (HC) pregnant women (n = 22). B, Myeloperoxidase (MPO)‐DNA complexes as markers of circulating NETs were measured in the same sera by MPO‐DNA ELISA. C, ELISA of MPO levels in sera of patients with APS (n = 22) or HCs. D, Neutrophil elastase (NE) was measured as another marker of NETs in sera of patients with APS (n = 22) or HCs using ELISA. *P < 0.05. Data are presented as the mean ± SD. ELISA, enzyme‐linked immunosorbent assay

Cell‐free DNA and NETs are increased in APS patients with thrombosis^19^ and other disease conditions, such as sepsis,^29^ small vessel vasculitis^23^ and thrombotic microangiopathy.^23^, ^30^ We thus examined these levels in the sera of pregnant women with APS (n = 22) and HCs (n = 22). Compared with HCs, APS sera had significantly greater levels of cell‐free DNA (256.70 ± 25.17 vs 184.70 ± 5.38 ng/mL, *P* < 0.05; Figure 1A). We further collected sera from 15 aPL‐positive patients without pregnancy morbidities and 18 aPL‐negative patients with pregnancy complications to determine cell‐free DNA levels. aPL‐positive patients with pregnancy morbidities showed significantly higher cell‐free DNA levels than those of the other three groups (Figure S1).

We also found higher levels of MPO‐DNA complexes, MPO and NE—credible markers of NETs—in APS sera than in HC sera (0.92 ± 0.10 vs 0.35 ± 0.06 OD; 223.70 ± 10.79 vs 117.00 ± 7.96 ng/mL; and 235.30 ± 24.45 vs 148.90 ± 11.46 ng/mL, respectively; all *P* < 0.05; Figure 1B‐D). Given that cell‐free foetal DNA is also released from the placenta in maternal sera,^31^ we performed correlation analysis between cell‐free DNA and MPO‐DNA in the sera of patients with APS and found a statistically significant correlation in these samples (Figure S2). This may indicate that the cell‐free DNA is at least partially neutrophil‐derived. Overall, the findings indicate that pregnant women with APS may have elevated levels of circulating cell‐free DNA and NETs compared with that of healthy pregnant women during early pregnancy.

### 3.2 Neutrophils from pregnant patients with APS are primed for NETosis

**FIGURE 2:**
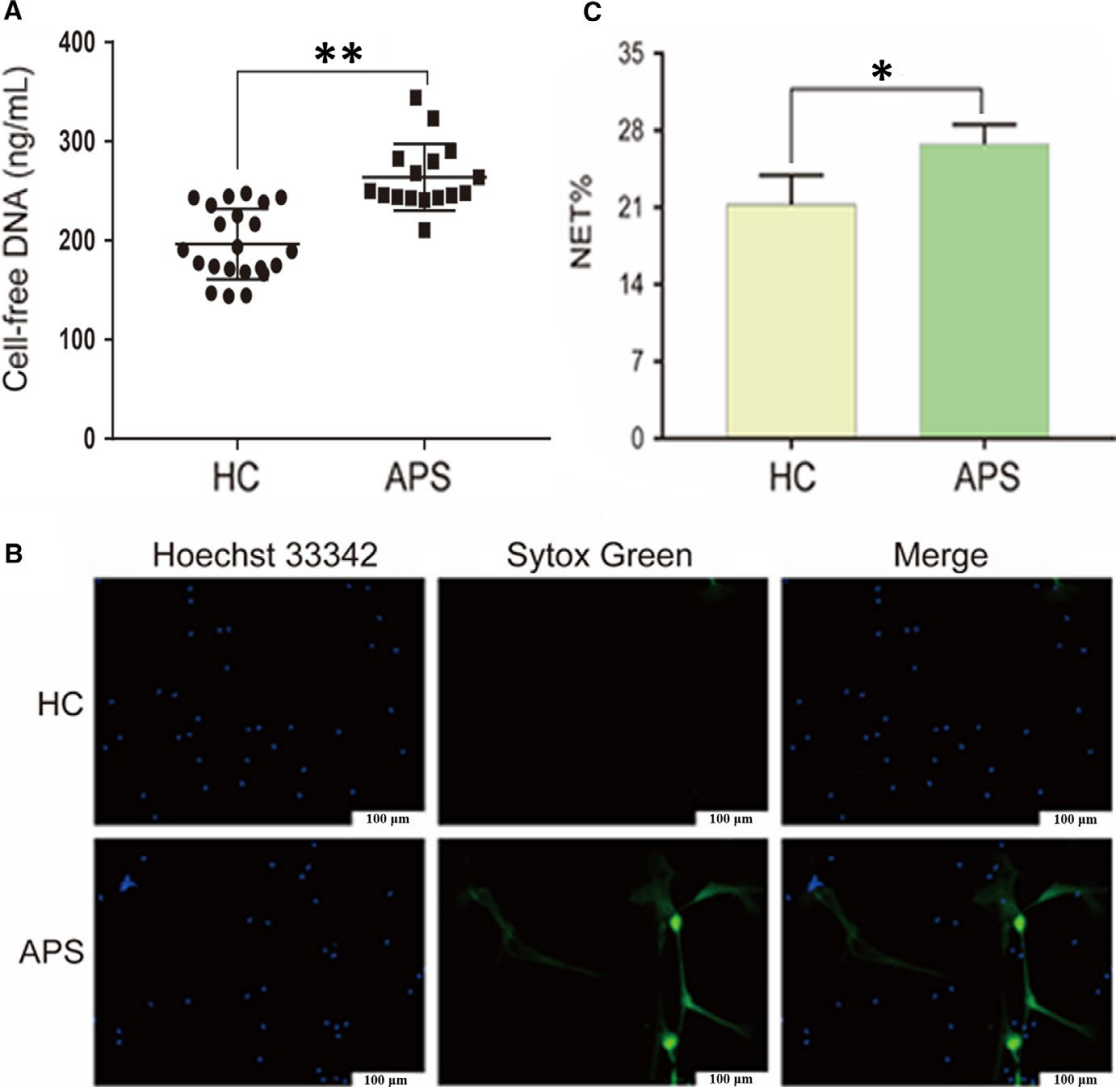
Neutrophils from patients with APS are primed to NETosis. Neutrophils were freshly isolated from HCs (n = 22) or pregnant women with APS (n = 16) and incubated in 96‐well plates without any in vitro stimulation for 2 h. A, Cell‐free DNA levels in the supernatant. B, Representative live‐cell images of HC and APS neutrophils. Extracellular DNA was detected with the cell‐impermeable dye SYTOX Green. Intracellular DNA was detected with the cell‐permeable dye Hoechst 33342. Scale bars = 100 microns. C, NET release was scored as presented in panel B. *P < 0.05, **P < 0.01. Data are presented as the mean ± SD (A) or SEM (C; at least three independent experiments). APS, antiphospholipid syndrome; HC, healthy control; NET, Neutrophil extracellular trap

Without specific in vitro stimulation, APS neutrophils displayed intensified spontaneous NET release compared with that of HC neutrophils (Figure 2A‐C). Moreover, cell‐free DNA in APS neutrophils was much higher than that in HCs (263.9 ± 8.44 vs 197.6 ± 7.85 ng/mL, *P* < 0.01; Figure 2A). To determine whether elevated cell‐free DNA results from NET release, the cell‐impermeable DNA dye SYTOX Green was used to monitor extracellular release of DNA from stimulated neutrophils, whereas the cell‐permeable DNA dye Hoechst 33342 was used to label intracellular DNA, followed by direct live‐cell imaging. The per cent of APS neutrophils forming NETs was significantly higher than that of HC neutrophils (26.75% ± 1.03% vs 21.25% ± 1.55%, *P* < 0.05; Figure 2B,C). The prolonged interval between peripheral blood collection and neutrophil isolation may affect cell‐free DNA levels (Figure S3). Therefore, we suggest that the blood should be processed within two hours of drawing from the donor.

### 3.3 IgG from APS sera stimulates neutrophils to release NETs

**FIGURE 3:**
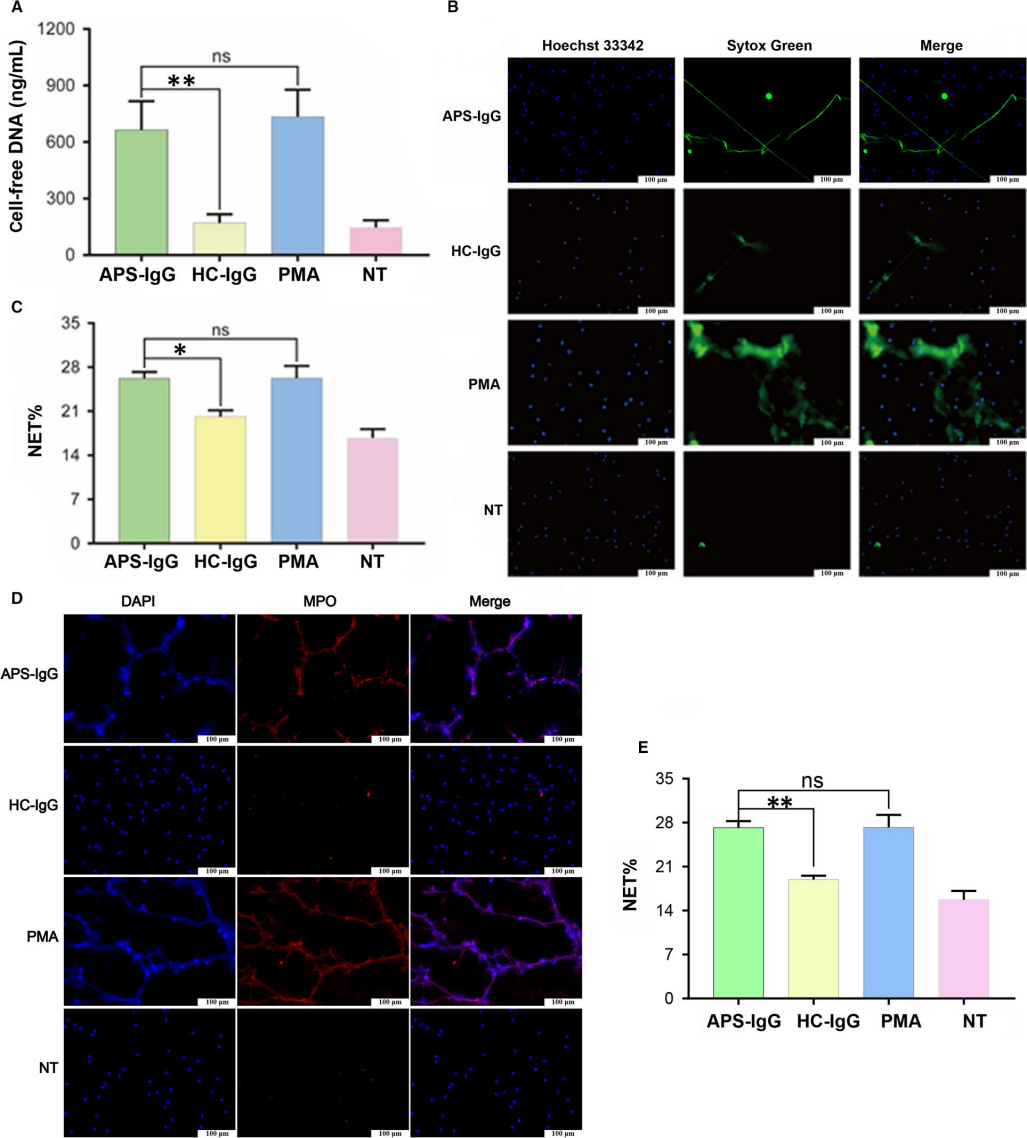
IgG from APS sera stimulates neutrophils to release NETs. Neutrophils isolated from healthy pregnant women were treated with APS‐IgG (15 µg/mL), HC‐IgG (15 µg/mL) and phorbol‐12‐myristate‐13‐acetate (PMA; 50 nmol/L) or left untreated (NT). A, Cell‐free DNA levels in the supernatant. B, Representative live‐cell images. SYTOX Green staining represents extracellular DNA; Hoechst 33342 staining represents intracellular DNA. Scale bars = 100 microns. C, NET release was scored as presented in panel B. D, Representative immunofluorescence images. NETs were co‐localized with MPO (red) and DNA (blue). Scale bars = 100 microns. E, NET release was scored as presented in panel D. Data are presented as the mean ± SEM of at least three independent experiments. *P < 0.05, **P < 0.01; ns, not significant. APS, antiphospholipid syndrome; HC, healthy control; MPO, myeloperoxidase; NET, Neutrophil extracellular trap

We incubated HC neutrophils with IgG isolated from APS sera (APS‐IgG) and HC sera (HC‐IgG). PMA, a known stimulus of NET formation, was used as a positive control. Neutrophils incubated with APS‐IgG released more cell‐free DNA than did neutrophils incubated with HC‐IgG (664.7 ± 50.88 vs 170.20 ± 15.42 ng/mL, *P* < 0.01; Figure 3A), which was confirmed by SYTOX Green staining (26.2% ± 0.60% vs 20.15% ± 0.58%, *P* < 0.05) and cell immunofluorescence (27.20% ± 0.60% vs 18.94% ± 0.36%, *P* < 0.01; Figure 3B‐E). Therefore, APS‐IgG significantly stimulates NET release compared with that of HC‐IgG.

### 3.4 APS‐IgG mediates NET release via ROS production

**FIGURE 4:**
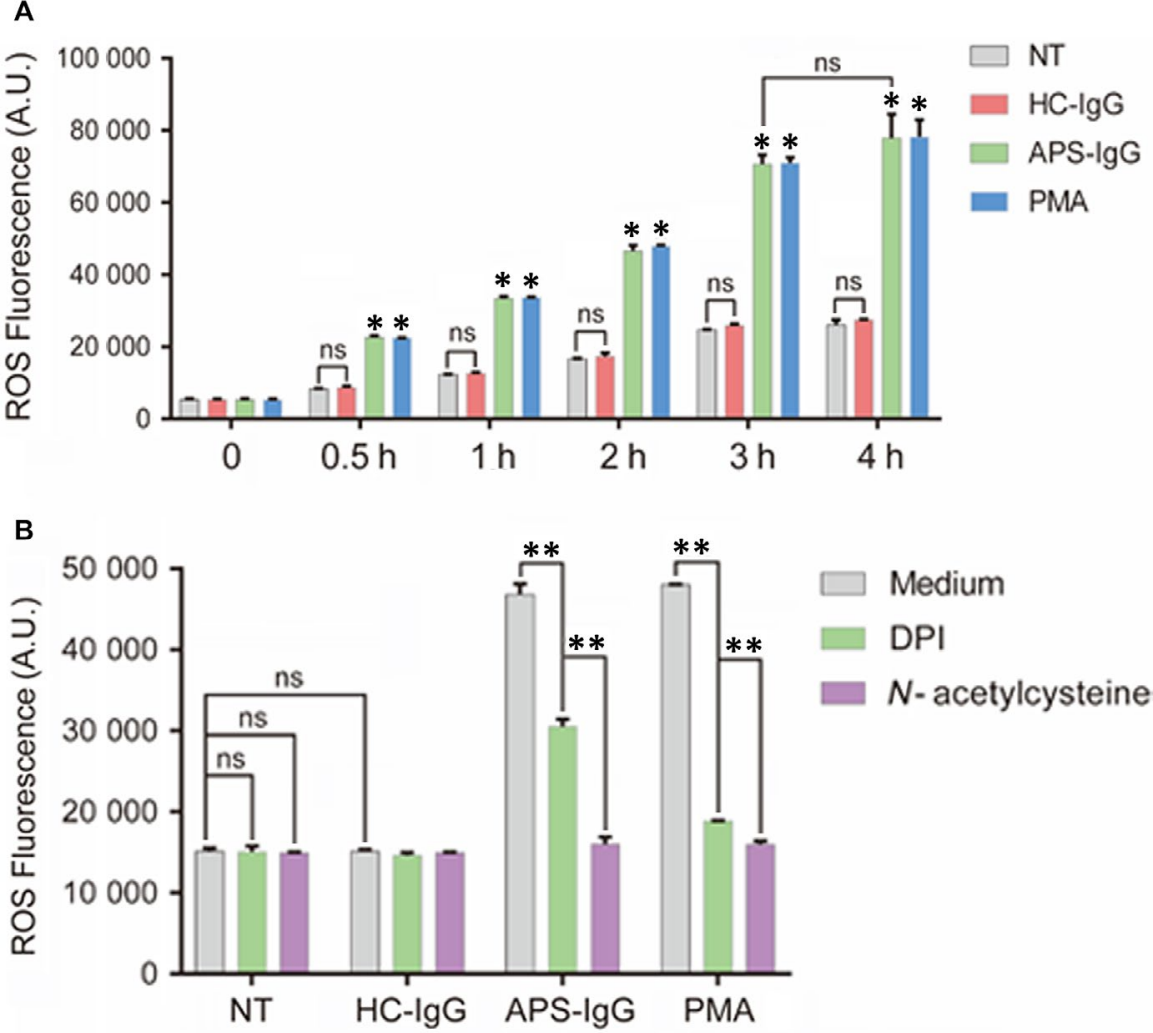
IgG from pregnant women with APS mediates NET release via reactive oxygen species (ROS) production. A, After stimulation with APS‐IgG (15 µg/mL), HC‐IgG (15 µg/mL) or PMA (50 nmol/L), neutrophils (3 × 10^4^/well) isolated from HCs were treated with DCFH‐DA at 0, 0.5, 1, 2, 3 and 4 h to determine ROS production. B, Neutrophils were pre‐treated with NADPH oxidase inhibitor diphenylene iodonium (DPI) and antioxygen N‐acetylcysteine, which notably suppressed ROS production, for 0.5 h, and then stimulated with APS‐IgG (15 µg/mL), HC‐IgG (15 µg/mL) or PMA (50 nmol/L) for 2 h. ROS production was determined via DCFH‐DA. Data are presented as the mean ± SEM of at least three independent experiments. *P < 0.05, **P < 0.01; ns, not significant. APS, antiphospholipid syndrome; HC, healthy control; NET, Neutrophil extracellular trap

To determine how APS‐IgG promotes NET release, we analysed ROS production by treating neutrophils from HCs with DCFH‐DA after stimulation with APS‐IgG, HC‐IgG or PMA. APS‐IgG accelerated ROS production in neutrophils in a time‐dependent manner and showed a similar effect to that of PMA (Figure 4A). After 4 hours of stimulation, ROS production reached a peak; however, there was no significant difference between 3 and 4 hours of stimulation (Figure 4A). Notably, both the NADPH oxidase inhibitor diphenylene iodonium (DPI) and antioxygen N‐acetylcysteine suppressed the ROS production triggered via APS‐IgG and PMA stimulation (Figure 4B). Pre‐treatment of neutrophils with DPI significantly decreased cell‐free DNA levels (Figure 6A), which was confirmed by live‐cell imaging (Figure 6B,C), highlighting the influence of ROS on these reactions. Our findings thus indicate that APS‐IgG stimulates NETosis through NADPH oxidase‐mediated ROS generation.

### 3.5 AKT, ERK1/2 and p38 MAPK pathways participate in NET release

**FIGURE 5:**
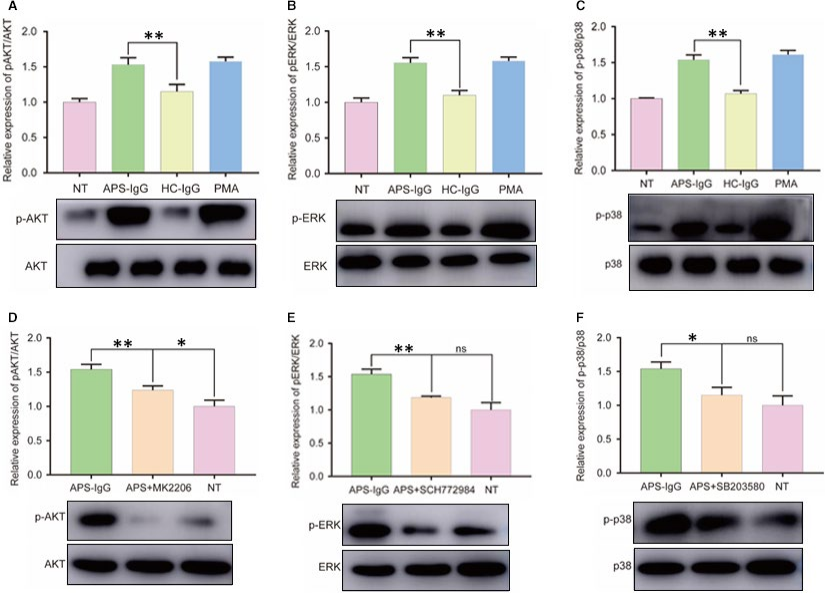
Effect of APS‐IgG and inhibitors on Akt, ERK1/2 and p38 MAPK phosphorylation in neutrophils. Neutrophils isolated from healthy pregnant women were treated with APS‐IgG (15 µug/mL), HC‐IgG (15 µg/mL) and PMA (50 nmol/L) or left untreated (NT) for 3 h. A‐C, Western blotting of p‐Akt (ser473), p‐ERK1/2 (Thr202/Tyr204) and p‐p38 MAPK (Thr180/Tyr182) expression. Neutrophils were separately pre‐treated with the inhibitors MK2206 (10 μmol/L), SCH772984 (10 μmol/L) or SB203580 (20 μmol/L) for 0.5 h, followed by 3 h of incubation with APS‐IgG (15 µg/mL) or complete medium (NT). D‐F, Western blotting of p‐Akt, p‐ERK1/2 and p‐p38 MAPK expression. Data are presented as the mean ± SEM of three independent experiments. *P < 0.05, **P < 0.01; ns, not significant. APS, antiphospholipid syndrome; HC, healthy control

As the AKT, ERK and p38 MAPK pathways are involved in PMA‐induced, ROS‐dependent NET release, phosphorylation of these molecules was assessed by Western blot analysis. We found that AKT, p38 MAPK and ERK1/2 phosphorylation was significantly increased in cells treated with APS‐IgG and PMA (Figure 5A‐C).

**FIGURE 6:**
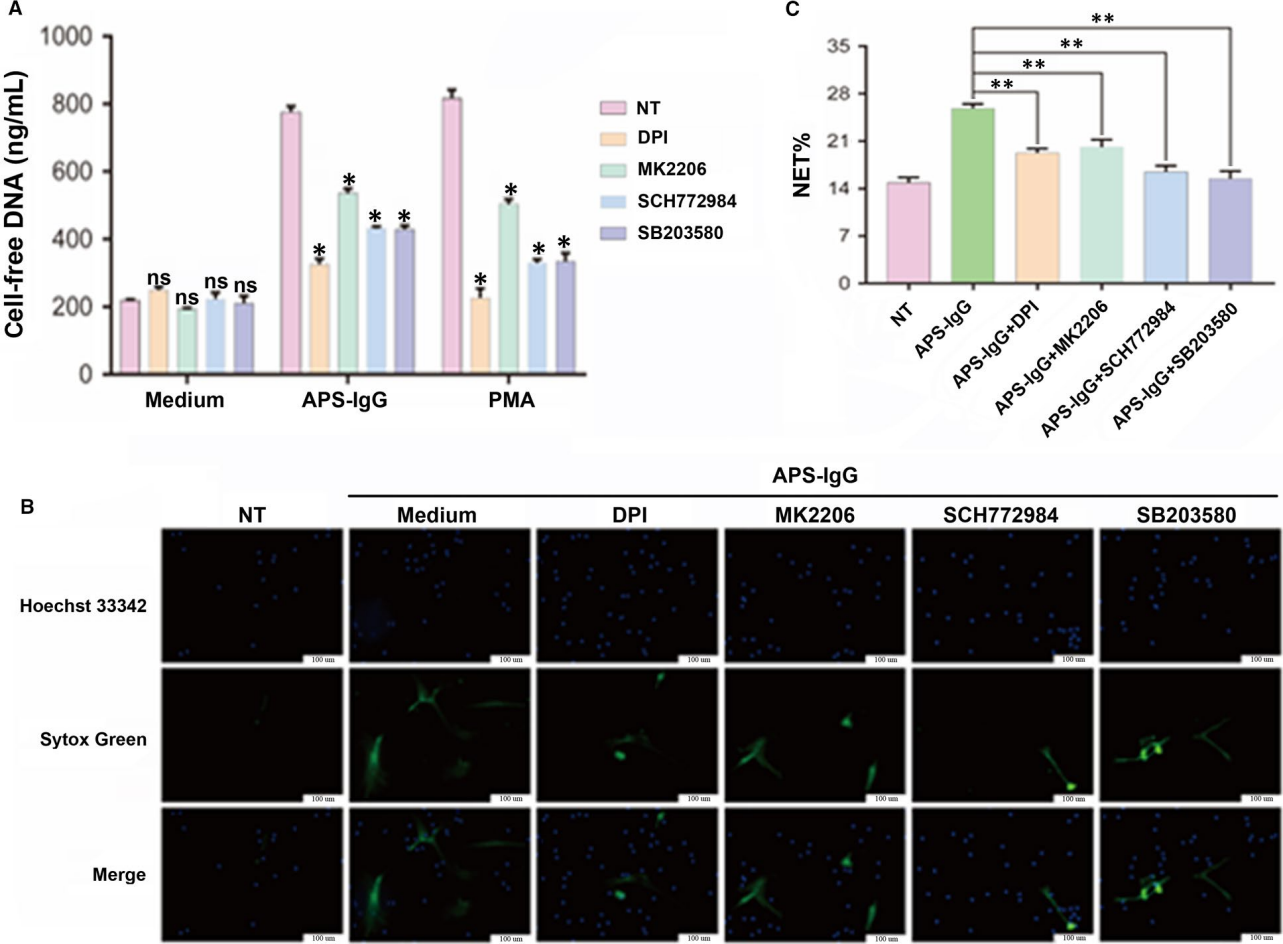
Effects of NADPH oxidase, AKT, ERK1/2 and p38 MAPK inhibitors on NET release. Freshly isolated neutrophils were separately pre‐treated with DPI (10 μmol/L), MK2206 (10 μmol/L), SCH772984 (10 μmol/L) or SB203580 (20 μmol/L) for 0.5 h, followed by incubation for another 3 h with APS‐IgG (15 µg/mL), PMA (50 nmol/L) or complete medium (no treatment; NT). A, Cell‐free DNA levels in the supernatant. B, Representative live‐cell images of NET release. SYTOX Green staining represents extracellular DNA; Hoechst 33 342 staining represents intracellular DNA. Scale bars = 100 microns. C, NET release was scored as presented in panel B. Data are presented as the mean ± SEM of at least three independent experiments. *P < 0.05, **P < 0.01; ns, not significant. APS, antiphospholipid syndrome; DPI, diphenylene iodonium; NET, Neutrophil extracellular trap

We next treated neutrophils cultured in the presence of APS‐IgG with specific AKT, ERK1/2 and p38 MAPK pathway inhibitors to assess their role in the induction of APS‐IgG‐mediated NET release. As shown in Figure 5D‐F, incubation with the relevant inhibitors for 30 minutes was sufficient to inhibit AKT, p38 MAPK and ERK1/2 phosphorylation. As expected, MK2206, SCH772984 and SB203580 decreased p‐AKT, p‐ERK and p‐p38 MAPK levels, respectively, whereas total AKT, ERK and p38 MAPK levels remained unaltered (Figure 5D‐F). MK2206, SCH772984 and SB203580 treatment had no effect on cell‐free DNA levels (Figure 6A). APS‐IgG combined with MK2206, SCH772984 and SB203580 treatment decreased cell‐free DNA levels (Figure 6A). Direct microscopic observation also yielded similar results (Figure 6B,C). These findings suggest that AKT, ERK1/2 and p38 MAPK pathway activation is required for NET release.

### 3.6 NETs triggered by APS‐IgG play a detrimental role in the invasion and migration of trophoblasts

**FIGURE 7:**
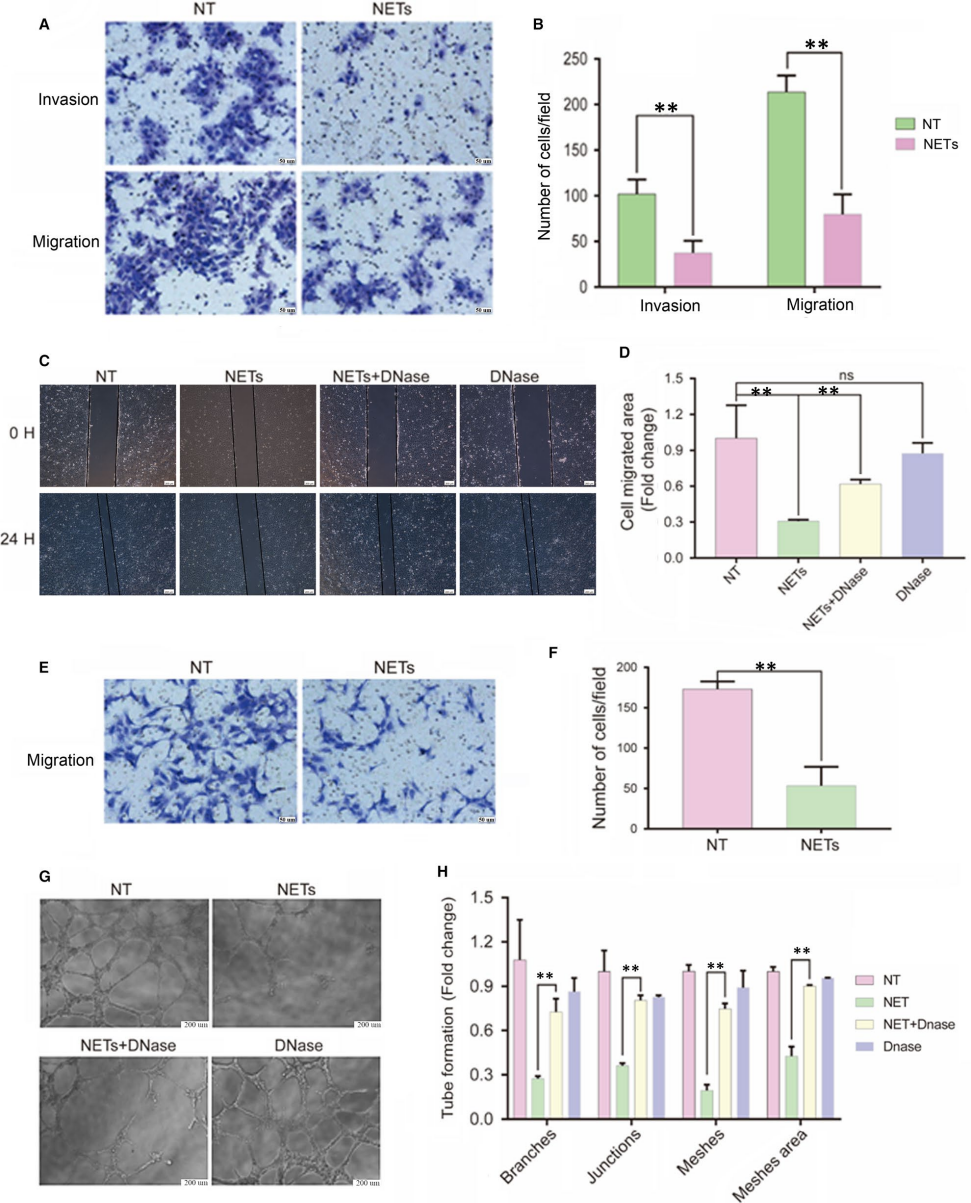
NETs induced by APS‐IgG have detrimental effects on the invasion and migration of trophoblasts as well as the migration and tube formation ability of HUVECs. HTR‐8/SVneo cells and HUVECs were treated with cell‐free NETs for 12 h. A, Representative images of Transwell invasion and migration assays. Scale bars = 50 microns. B, Values represent mean ± SEM (n = 3 independent experiments) of the number of cells per field. C, Representative images of wound‐healing assays at 0 h and 24 h. Scale bars = 200 microns. D, Values represent mean ± SEM (n = 3 independent experiments) of cell migrated area. For the tube formation assay, an additional group consisted of HUVECs incubated with DNase I (10 U/mL, 37℃ and 1 h). E, Representative images of Transwell migration assay. Scale bars = 50 microns. F, Values represent mean ± SEM (n = 3 independent experiments) of the number of cells per field. G, Representative images of tube formation assay at 8 h. Scale bars = 200 microns. H, Values represent mean ± SEM (n = 3 independent experiments) of the number of tube branches, junctions, meshes and mesh area. **P < 0.01; ns, not significant. APS, antiphospholipid syndrome; HC, healthy control; NET, Neutrophil extracellular trap

To analyse the effects of NETs on the human first‐trimester extravillous trophoblast cell line HTR‐8/SVneo, cell‐free NETs prepared in vitro were used. Proper trophoblast migration and invasion are important for normal placentation, which is important in establishing blood flow between the mother and embryo. A Transwell assay revealed that HTR‐8/SVneo cell invasion and migration are inhibited by NET treatment compared with that of control (no treatment, NT; Figure 7A,B); the difference between the invasion and migration assays was that HTR‐8/SVneo cells were incubated on BD Matrigel in the invasion assay. Incubation with APS‐IgG‐stimulated NETs significantly decreased the number of invading or migrating HTR‐8/SVneo cells from the upper chamber to the lower chamber (invasion: NETs 37.33 ± 7.69 vs NT 102.00 ± 9.07, *P* < 0.01; migration: NETs 79.67 ± 12.81 vs NT 213.70 ± 10.41, *P* < 0.01). Wound‐healing assays corroborated the results and showed that this inhibition could be partly interrupted by DNase I (Figure 7C,D). The cell migration area was reduced when HTR‐8/SVneo cells were incubated with APS‐IgG‐stimulated NETs, which was partly improved when cells were treated with NETs and DNase I simultaneously. The effect of DNase I on trophoblasts treated with PMA‐induced NETs was similar (Figure S4A‐D), whereas DNase I alone had the same effect as no treatment. When treated with HC‐IgG‐induced NETs, trophoblast migration and invasion capacity did not show the same changes as when treated with APS‐IgG‐induced NETs (Figure S5A‐D).

### 3.7 **APS‐IgG**‐**induced NETs influence the migration and tube formation ability of HUVECs**

Endothelial cells play a decisive role in maintaining homeostasis during the first trimester, which is necessary for sustaining normal pregnancy; thus, we examined the effects of NETs on HUVECs and found that NETs inhibited HUVEC migration (53.33 ± 13.54 vs 173.00 ± 5.51, *P* < 0.01; Figure 7E,F), as demonstrated by Transwell assays. We also evaluated the effects of APS‐IgG‐induced NETs on the angiogenic ability of HUVECs by performing a tube formation assay. Exposure to NETs reduced tube formation, as demonstrated by the decreased number of tube branches, junctions, meshes and mesh areas. These inhibitory effects were reversed, in part, when DNase I was added (Figure 7G,H). The effects of DNase I on PMA‐induced NETs were similar to its effects on APS‐IgG‐induced NETs (Figure S4E‐H). Moreover, when treated with HC‐IgG‐induced NETs, HUVEC migration and tube formation abilities did not show the same changes as when treated with APS‐IgG‐induced NETs (Figure S5E‐H).

## 4 DISCUSSION

Thrombosis and pregnancy‐related morbidity are the main clinical manifestations of APS.^1^ With advances in research, the question of whether obstetric and vascular APS are the same or different diseases has arisen.^32^ Defective placentation is considered a distinct characteristic of pregnancy complications, such as miscarriage and pre‐eclampsia^33^; however, the induction of defective placentation and mechanisms involved remains unclear. In obstetric APS, it is known that pathogenic aPLs, recognizing β2GPI, target the placenta, leading to adverse pregnancy outcome.^34^ A recent study showed that aPLs inhibit negative regulators of placental trophoblast Toll‐like receptors and inflammasome signalling to trigger an inflammatory response.^4^

In the current study, we first detected cell‐free DNA and NET marker levels in the sera of pregnant women with or without APS. The effects of APS‐IgG on the capacity of neutrophils to release NETs from pregnant women with APS were first explored, after which the influence of cell‐free NETs on trophoblast and HUVEC function was investigated. We also examined the pathways involved in APS‐IgG‐induced NETosis.

Neutrophils are the first line of defence in the body's innate immune system and play important roles via different mechanisms, such as NETosis,^35^ which is the process of releasing NETs. However, NETs are involved in the pathogenesis of many autoimmune and inflammatory diseases; for instance, the protein content in NET granules may be the source of antigens for pathogenic autoantibodies in ANCA‐AAV.^36^ Inflammatory cytokines, such as IL‐17 and TNF‐a, were also shown to induce NETosis in RA cases.^37^ Moreover, enhanced NET formation was observed in patients with SLE, which is connected to both autophagy and defective clearance of NETs.^38^, ^39^ Marder et al^40^ evaluated placentas from 35 pregnancies and found that the number of neutrophils and NETs was elevated in the placenta of patients with SLE and pre‐eclampsia. This suggests that NETs play a role in placenta‐mediated pathological pregnancies. Similarly, we showed in the present study that NET markers were significantly increased and NET degradation decreased in obstetric APS compared with healthy pregnant women. We also found that IgG isolated from pregnant women with APS triggered neutrophils to release NETs, which is in accordance with the effects of IgG from patients with vascular APS on NET release.^19^

NETosis is associated with the function of NADPH oxidase, which produces the ROS critical for NET generation.^41^ Our findings demonstrated that APS‐IgG induces ROS production and that NADPH oxidase inhibitors (DPI and antioxygen N‐acetylcysteine) can suppress the NET release and ROS production triggered via APS‐IgG and PMA, indicating that aPLs stimulate NETs by triggering ROS production. Therefore, ROS scavengers, such as N‐acetylcysteine and DNase, represent a potential therapeutic strategy for treating obstetric APS. Indeed, ROS scavengers have already been used and their efficacy proven for reducing circulating NETs in SLE.^42^, ^43^

Papayannopoulos^44^ demonstrated that the amount of NETs and the mechanism of NET formation vary according to the stimulus. Hakkim et al^45^ were the first to reveal the role of RAF‐MEK‐ERK signalling pathways in PMA‐induced NET formation. The MAPK pathway regulates cellular events by transferring external and internal signals. ERK1/2 and p38 MAPK are the main constituents of the MAPK family.^46^ In our study, APS‐IgG was found to activate ERK1/2 and p38 MAPK, in addition to promoting phosphorylation of the AKT pathway. Interestingly, AKT, ERK1/2 and p38 MAPK inhibitors only partly inhibited APS‐IgG‐induced NETosis, demonstrating that although AKT, ERK1/2 and p38 MAPK were necessary for aPL‐induced ROS production and NET formation, there are other pathways taking part in aPL‐induced NET formation. The signalling pathway involved in pathogenesis is likely to resemble that of PMA‐induced NET release, instead of lipopolysaccharide‐induced NET release.^47^ The relationship between AKT, ERK1/2 and p38 MAPK in aPL‐induced NET formation and other pathways needs further investigation.

NET components, such as DNA and histones, promote thrombosis in different diseases.^48^ For instance, the DNA backbones of NETs are capable activators of factor XII because of its negatively charged surface.^49^ Meanwhile, histones may accelerate coagulation via promotion of platelet activation, aggregation and thrombin generation.^50^, ^51^ Besides thrombosis, NETs have been shown to trigger endothelial and epithelial cytotoxicity and induce endothelial dysfunction by decreasing cell proliferation and increasing cell apoptosis.^14^, ^25^, ^52^ Recently, NETs were found to play an important role in bladder cancer treatment by attacking tumour cells.^18^ In the present study, we further found that NETs decrease the invasive and migratory abilities of trophoblasts and have a negative effect on the migration and tube formation ability of HUVECs. Therefore, we suggest that NETs contribute to pregnancy morbidity by directly decreasing cell migration and invasion and tube formation. As DNase I partly limited the negative effects of NETs on trophoblast migration and endothelial cell tube formation, stretches of DNA in APS‐IgG‐induced NETs may be responsible for negatively affecting trophoblasts and endothelial cells. This finding prompts further investigation to identify whether DNase I activity is decreased or whether NETs are resistant to DNase I in pregnant women with APS.

Taken together, our results indicate that NET markers are increased in pregnant women with APS and that NETs may potentially contribute to defective placentation. Further studies are needed to evaluate the effect of NETs on placentation in vivo, which may reveal the underlying mechanisms and role of NETosis in obstetric APS pathogenesis. Research in this direction may provide prognostic markers or potential new targets for the treatment of this disease.

## CONFLICT OF INTEREST

The authors confirm that there are no conflicts of interest.

## AUTHOR CONTRIBUTION

Xietong Wang designed the study. Yuan Lu and Yan Dong performed the research. Yuan Lu and Yu Xia analysed the data and wrote the paper. Yan Zhang, Di Shen, Xiyao Wang, Ruxiu Ge and Meihua Zhang collected clinical blood and umbilical cord samples. All authors have reviewed and approved the manuscript.

## Supporting information

Fig S1

Fig S2

Fig S3

Fig S4

Fig S5

Table S1

## Data Availability

The data that support the findings of this study are available from the corresponding author upon reasonable request.
