# Supplementary figures and images for: Antiphospholipid antibody‐activated NETs exacerbate trophoblast and endothelial cell injury in obstetric antiphospholipid syndrome
Source: J Cell Mol Med. 2020 May 5;24(12):6690–703. doi: 10.1111/jcmm.15321 (PMC7299718; doi:10.1111/jcmm.15321)

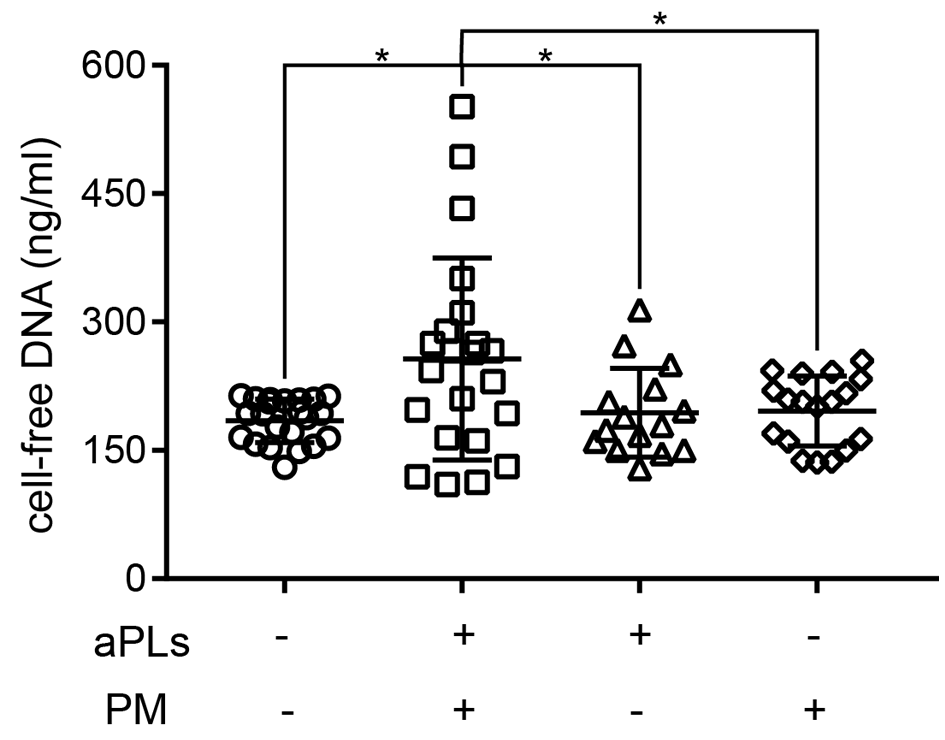

Supplement: Supplementary file 1 — Fig S1 [file JCMM-24-6690-s001.tif]

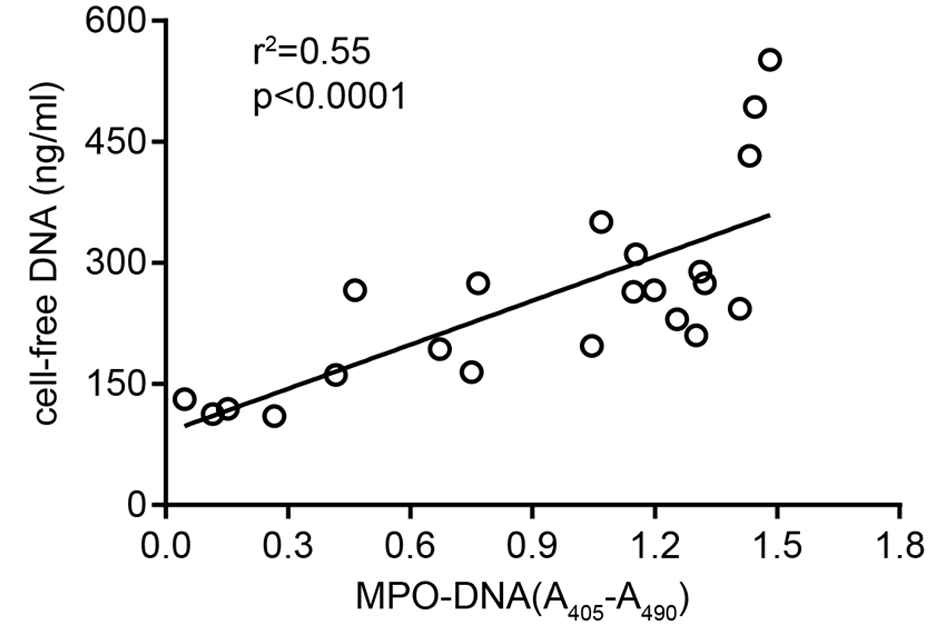

Supplement: Supplementary file 2 — Fig S2 [file JCMM-24-6690-s002.tif]

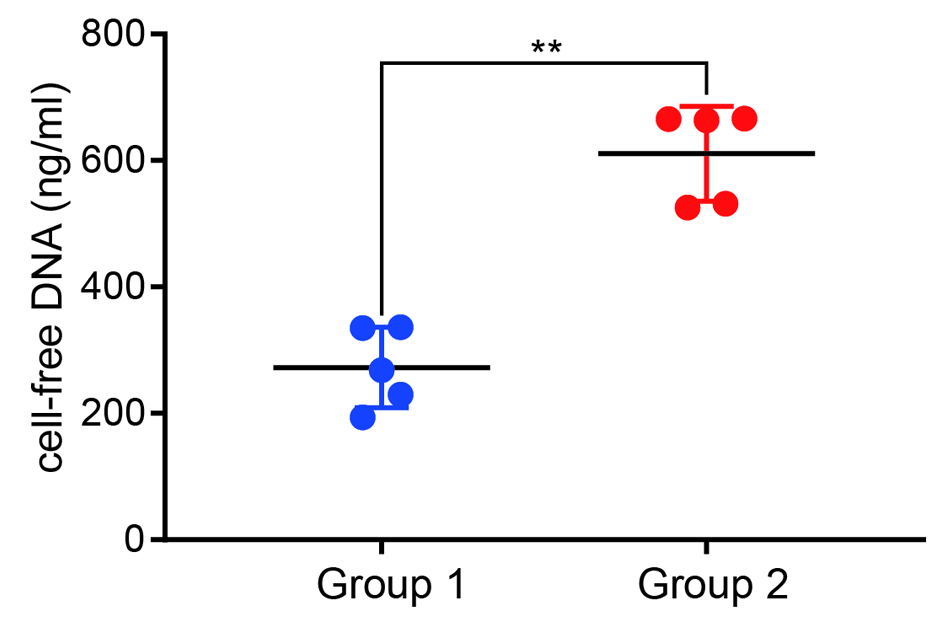

Supplement: Supplementary file 3 — Fig S3 [file JCMM-24-6690-s003.tif]

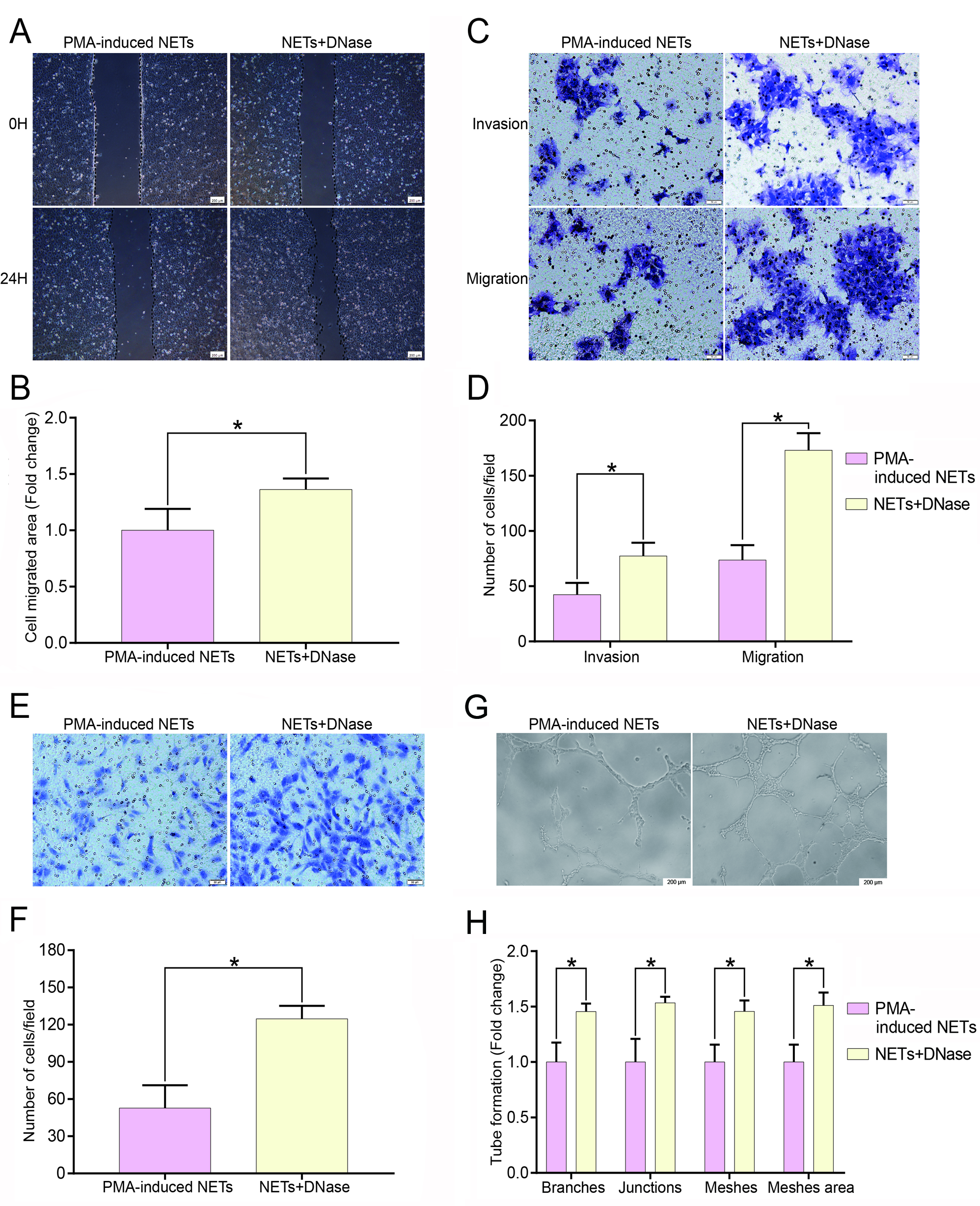

Supplement: Supplementary file 4 — Fig S4 [file JCMM-24-6690-s004.tif]

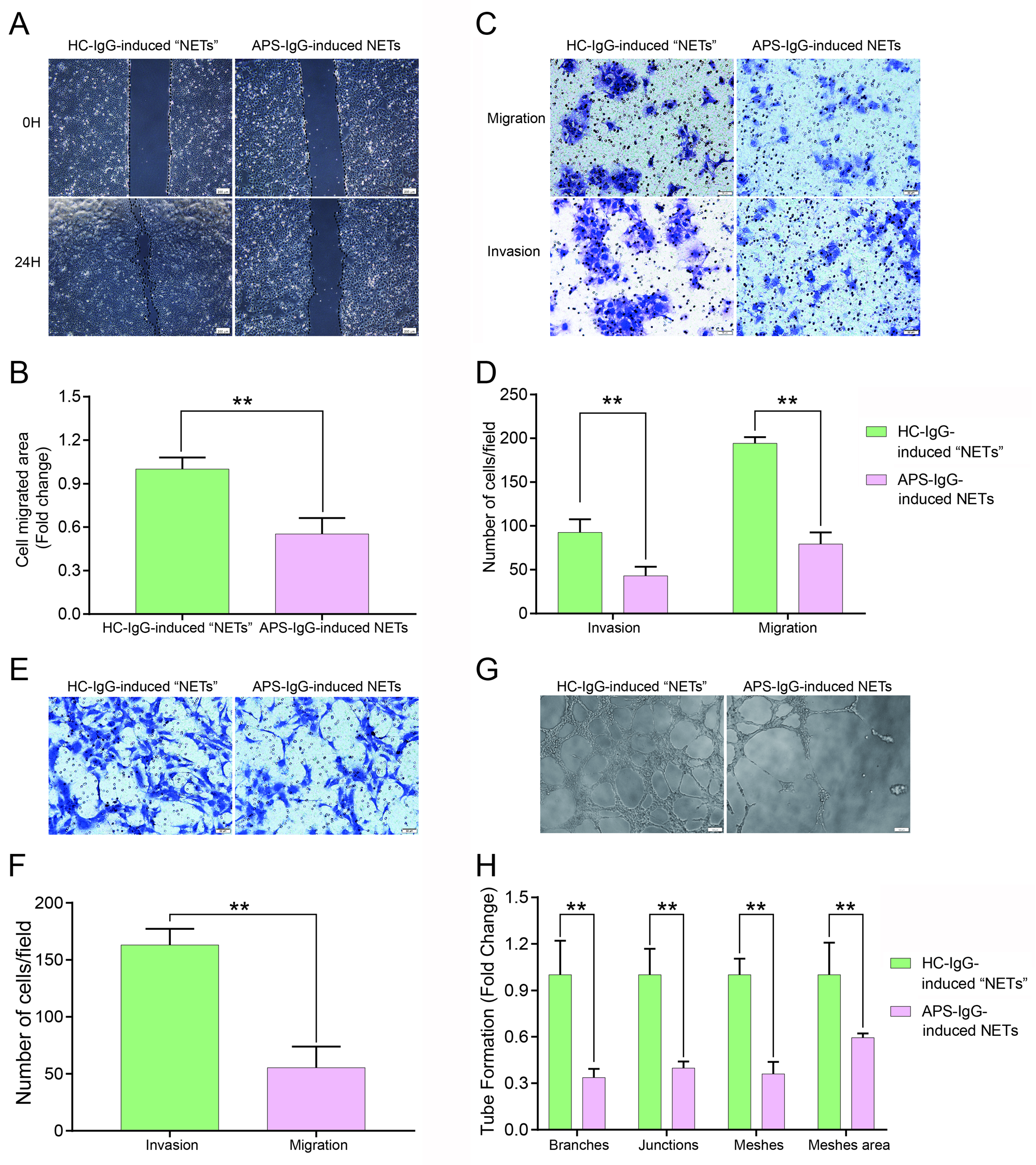

Supplement: Supplementary file 5 — Fig S5 [file JCMM-24-6690-s005.tif]
