# Supplementary material for: Antiphospholipid antibody‐activated NETs exacerbate trophoblast and endothelial cell injury in obstetric antiphospholipid syndrome
Source: J Cell Mol Med. 2020 May 5;24(12):6690–703. doi: 10.1111/jcmm.15321 (PMC7299718; doi:10.1111/jcmm.15321)
Supplement: Supplementary file 6 — Table S1 [file JCMM-24-6690-s006.docx]

Supplementary Table 1. Gestation weeks of patients with APS and HCs

| Number | APS  (gestational week) | HC  (gestational week) |
| --- | --- | --- |
| 1 | 11.71 | 12.71 |
| 2 | 12.57 | 11.86 |
| 3 | 12.00 | 12.71 |
| 4 | 12.29 | 12.86 |
| 5 | 12.43 | 12.71 |
| 6 | 11.00 | 13.29 |
| 7 | 11.43 | 12.71 |
| 8 | 12.14 | 11.71 |
| 9 | 11.14 | 11.86 |
| 10 | 11.29 | 13.86 |
| 11 | 11.57 | 11.14 |
| 12 | 12.71 | 12.57 |
| 13 | 12.43 | 12.57 |
| 14 | 12.57 | 12.43 |
| 15 | 11.86 | 11.14 |
| 16 | 11.71 | 12.14 |
| 17 | 12.00 | 12.00 |
| 18 | 13.00 | 12.57 |
| 19 | 13.14 | 13.43 |
| 20 | 13.29 | 13.14 |
| 21 | 13.57 | 13.00 |
| 22 | 12.43 | 11.86 |

APS, antiphospholipid syndrome; HC, healthy control
